# Supplementary figures and images for: A software ecosystem for brain tractometry processing, analysis, and insight
Source: PLoS Comput Biol. 2025 Aug 14;21(8):e1013323. doi: 10.1371/journal.pcbi.1013323 (PMC12373284; doi:10.1371/journal.pcbi.1013323)

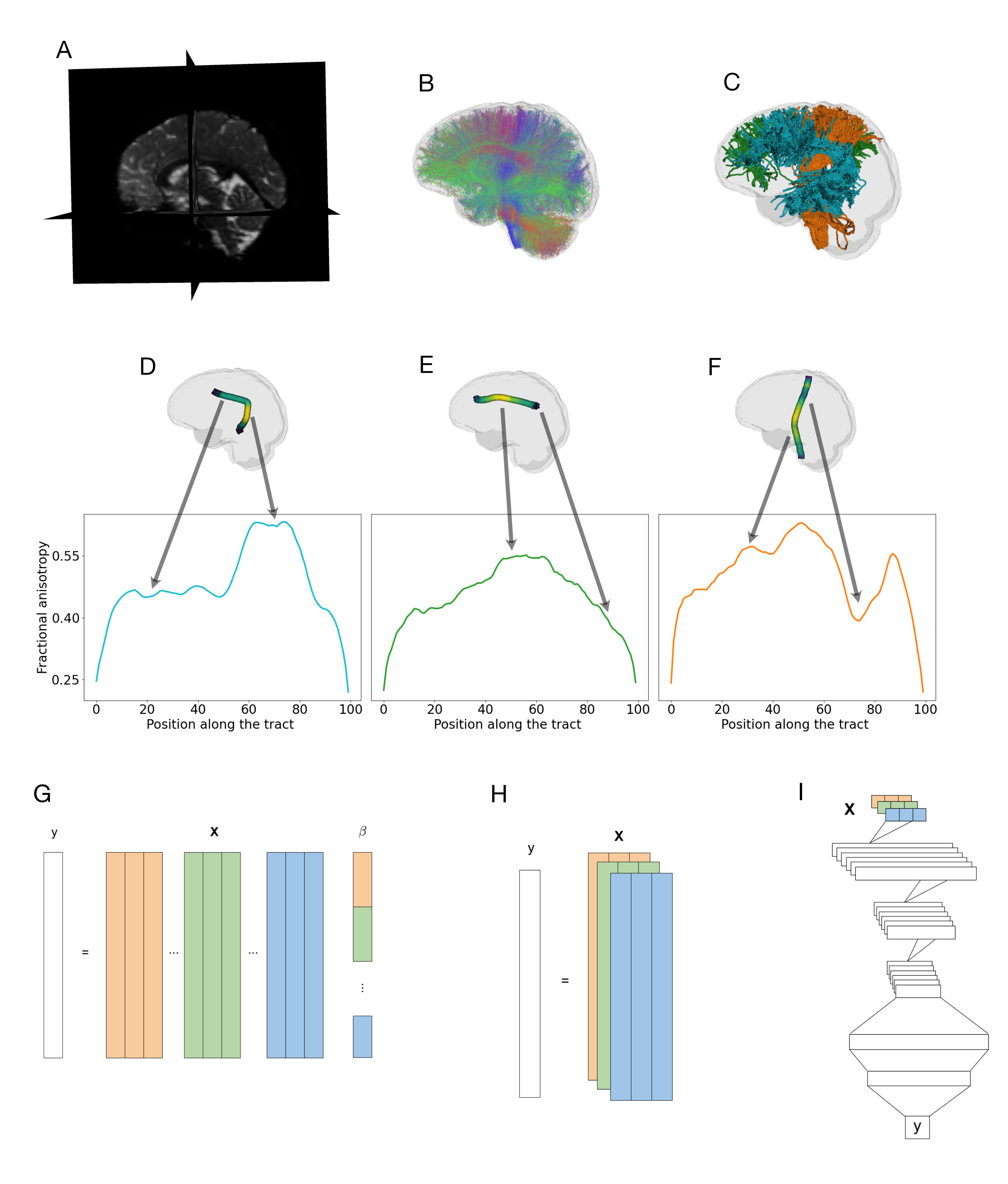

Supplement: S1 Fig — (A) Diffusion MRI (dMRI) data from the HBN-POD2 sample is used as input to the algorithm. (B) In the initial step, whole-brain tractography is conducted (C) Major white matter pathways are identified based on the trajectory of the streamlines. Here, the arcuate fasciculus (blue), cingulum cingulate (green) and corticospinal tract (orange) are displayed as an example. (D-F) Features of the tissue along the length of each tract are extracted from the image into a “tract profile”. For each of the identified white matter pathways, the tract profile is extracted as a one-dimensional vector of numbers. (G) The brain age prediction problem set up as a linear model. Here y is a column vector of the ages of the different subjects in the sample, X is a matrix with each row containing the dMRI tract profiles of one subject. Features from each tract are color-coded as before. For example, all of the 100 values of the FA in the arcuate fasciculus may be stored in the columns indicated in blue. (H) To use as input to NN algorithms, the data in X is transformed into a three-way tensor, with one dimension associated again with the subjects, another dimension associated with the position along the length of each tract (from 1 to 100) and the last dimension (known as the “channel” dimension in analysis of images) associated with the different tracts and metrics. (I) Schematic of a multi-layered neural network where the data from one subject (i.e., one row of the three-way tensor) is provided as input and the output is compared to the age of the subject (y) and the error can be propagated back to adjust the parameters of the network. (PNG) [file pcbi.1013323.s001.png]

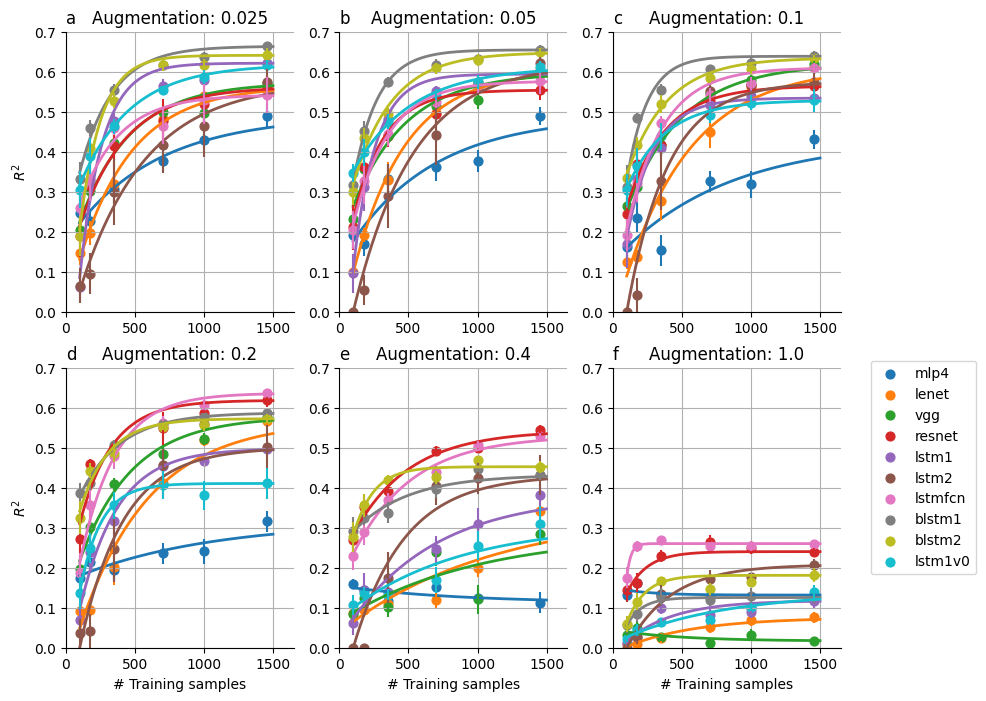

Supplement: S2 Fig — Data augmentation introduces random noise to each sample of the training data in each batch of training. This method can help NN algorithms with a large number of parameters generalize better, by preventing the memorization of the samples in the training set. When augmentation levels grow very large, however, the signal in the data is overwhelmed by the noise that is added in augmentation, and the algorithm can no longer learn. In our data, we found that augmentation can have dramatic effects on algorithm performance in the brain age prediction task. For example, the resnet NN algorithm, which had poor R2 in the augmentation-free condition, reaches parity with the baseline model at relatively high augmentation levels (R2=0.62±0.016 standard error of the mean (SEM), red curves). The lstmfcn NN, which also performs poorly with no augmentation, reached even higher R2 than the baseline model with high levels of augmentation (R2=0.64±0.009 SEM, pink curves). However, at these higher levels of augmentation, the data requirements of these two models also increases. Algorithms that were similar in their performance to the baseline in the absence of augmentation improve slightly with the introduction of small amounts of augmentation. For example, the highest R2 reached by any model in these experiments is reached by the blstm1 model at a low value of augmentation (R2=0.66±0.01 SEM, gray curves). The relatively-simple mlp4 model architecture that does not perform very well in the absence of augmentation, only becomes worse with the introduction of augmentation (blue curves). Further quantification of these trends is laid out in S3 Fig, S4 Fig, S5 Fig. (PNG) [file pcbi.1013323.s002.png]

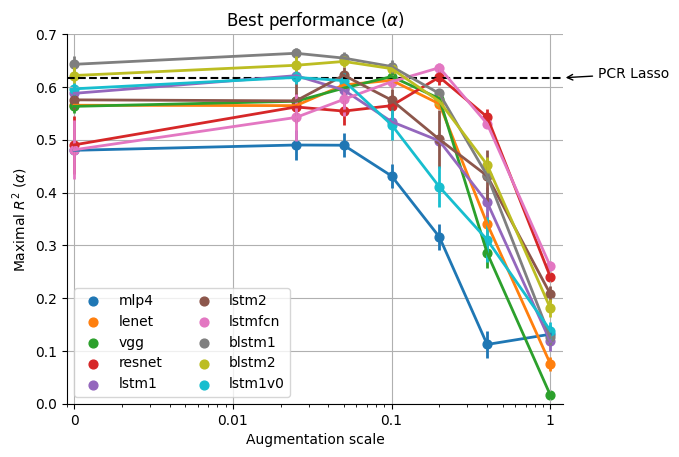

Supplement: S3 Fig — The black dashed line indicates the R2 of the linear baseline model (PCR Lasso). As seen in S2 Fig, some algorithms only decrease in their performance with increased augmentation (e.g., mlp4, blue curve), but many of the NN algorithms improve their performance with increased augmentation, with some (e.g., resnet, red curve and lstmfcn, pink curve) reaching parity of R2 with PCR Lasso at higher levels of augmentation. At higher levels of augmentation the noise added to the measurements overwhelms all of the useful signal for training and all algorithms perform poorly. (PNG) [file pcbi.1013323.s003.png]

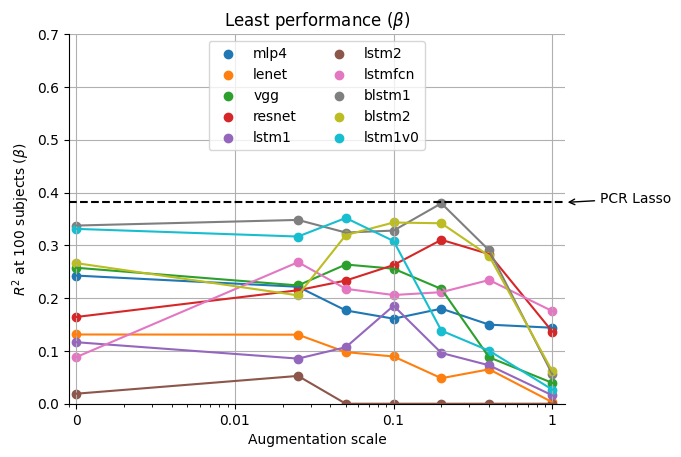

Supplement: S4 Fig — The resilience of the algorithms is quantified for cases where only limited data is available. Almost none of the algorithms, across all augmentation levels, are as resilient to smaller training data as PCR Lasso (dashed line). (PNG) [file pcbi.1013323.s004.png]

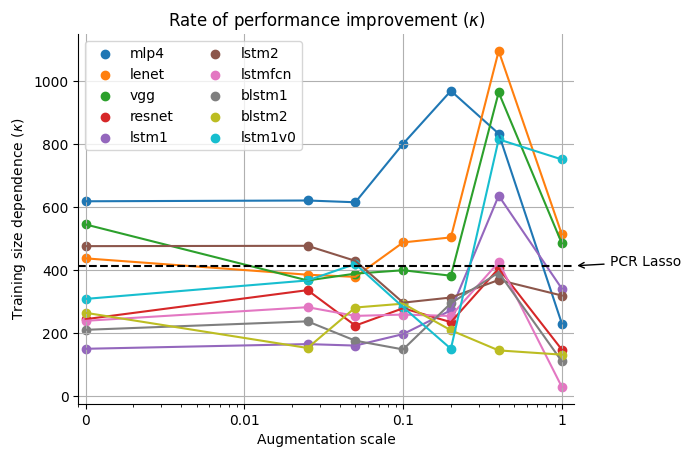

Supplement: S5 Fig — Here, smaller values indicate more favorable performance (i.e., the algorithm requires a smaller sample size to reach ∼63% of its best performance). Several of the algorithms show improved resilience to small sample sizes, relative to the linear baseline (dashed line, PCR Lasso), even under conditions where performance is better than PCR Lasso (e.g., compare blstm1 curve with S1 Fig) (PNG) [file pcbi.1013323.s005.png]
