# Supplementary material for: A software ecosystem for brain tractometry processing, analysis, and insight
Source: PLoS Comput Biol. 2025 Aug 14;21(8):e1013323. doi: 10.1371/journal.pcbi.1013323 (PMC12373284; doi:10.1371/journal.pcbi.1013323)
Supplement: Attachment — Submitted filename: response_to_reviewers.pdf [file pcbi.1013323.s006.pdf]

We thanks the reviewers for taking the time to review our article. We addressed all of their comments and suggestions in our response below. In addition, we have reformatted the article based on requests from the PLoS Computational Biology production team.

In our responses, reviewer comments are preserved in full in black *italic* font, and our responses are typeset in blue. Line numbers mentioned below refer to the "clean" version of the article (where changes are not marked up.)

# 1 Additional Editor Comments:

> *This manuscript has been reviewed by two external experts, and, while mostly positive, there are some clarifications and comments to be addressed before suitable for acceptance. First, please ensure that the code base is usable and has adequate documentation, and second, please make sure to clarify what is new in this manuscript - even if it is to provide comprehensive description of software. Thank you!*

In response to reviewer comments and this comment from the editor, we have made several changes to the manuscript. We have also revised the computational infrastructure that we provided for replication of the work. In particular, we have clarified in our manuscript that it describes an entirely revised new version of previously-published methods in a manner that increases interoperability and enhances its functionality. This is emphasized in new text added to the Introduction (lines 41-46) Materials and Methods (lines 59-62) and Discussion (lines 685-688).

# 2 Reviewer #1:

> *This manuscript presents a comprehensive software ecosystem for tractometry analysis based on diffusion MRI, centered on the widely recognized pyAFQ platform. The authors—well-established figures in the field of diffusion imaging and neuroimaging software development—have consolidated multiple efforts into a cohesive, extensible, and open-source pipeline. The manuscript is clearly written, the scope aligns well with the goals of a Software article, and the work has the potential for significant impact given the increasing demand for reproducible and scalable tractometry pipelines.*
>
> *Strengths:*
>
> *pyAFQ and its ecosystem are already well adopted by the diffusion MRI community.*
>
> *The software is open-source, well-maintained, and integrates modern standards (e.g., BIDS, QSIPrep), with a strong emphasis on reproducibility.*
>
> *The benchmarking and performance evaluations are thorough and demonstrate meaningful improvements.*
>
> *Minor Suggestions for Revision (optional):*
>
> *To be honest, this manuscript is already suitable for acceptance. The following are entirely optional suggestions that may help broaden the accessibility and impact of the work, especially for potential users and readers unfamiliar with pyAFQ. I fully understand if the authors choose not to incorporate these suggestions.*

Thank you for your positive assessment of the manuscript and for highlighting these strengths

> *Clarify the Machine Learning Section: The manuscript includes a detailed description of machine learning models (e.g., MLP, CNN, RNN), but the rationale behind model selection and optimization could be further explained. For instance, why did certain architectures*

*(e.g., BLSTM) outperform others? A brief explanation—even qualitative—would help readers appreciate the modeling choices, especially those less familiar with sequence modeling. A small demonstration or case example would also make this section more engaging and informative.*

We have added more rationale for each model type (lines 301-303,309-313,328-332,341-342), and we expand on the interpretation of the performance advantage of the BLSTM model (lines 726-730). The code examples that are provided in code ocean and neurolibre include more vignettes of machine learning applications. We added a sentence referring the readers to these examples in the Discussion (lines 740-745, 837-844) and Introduction (lines 46-51).

*Expand on Use Case Examples: While age prediction is a strong example, the manuscript could benefit from highlighting additional applications—particularly those in clinical or longitudinal research. Given the availability of disease-related datasets on platforms like OpenNeuro, demonstrating how pyAFQ could be applied to neurological disorders (e.g., ALS, SC2, Stroke...etc.) may further enhance its relevance and utility.*

We agree that showing the utility of these tools to disease is important. The manuscript already includes a section that uses data from participants with ALS ("Statistical modeling"), and our previous work has demonstrated the application of AFQ-Insight regularized regression models to this data (Richie-Halford et al., 2021). However, this dataset is far too small to apply neural networks to. Moreover, it is quite hard to find a dataset of suitable size to apply these methods. Nevertheless, in a previous study, we used some of the methods described here to study a large sample of individuals with glaucoma from the UK Biobank (Kruper et al., 2024), and we refer to this study in the Discussion. We feel that an additional demonstration of the application of these methods to a clinical dataset goes well beyond the scope of a software paper, and would constitute an empirical study that may better stand on its own.

*Clarify Statistical vs. Machine Learning Approaches: The transition between statistical analysis and machine learning modeling felt a bit abrupt. Including a brief summary or schematic contrasting the two (e.g., interpretability vs. predictive power) could help orient readers to the different goals and outcomes of each approach.*

We have added a few sentences at the beginning of the section titled "Machine learning and predictive modeling" to ease this transition (lines 582-588). We also pick up on the theme of predictive power vs. interpretability when Discussing these methods, line 739

*Minor Clarifications: Please clarify whether all benchmarking datasets (e.g., HBN, ALS) were processed exclusively through QSIPrep, or if other preprocessing pipelines were also considered.*

That is a good point. The HBN dataset was processed through qsiprep but the ALS dataset was processed using other tools. We consider the multiple potential entry points into the software to be an advantage.

*Consider including a concise table summarizing the tools in the ecosystem (e.g., pyAFQ, Tractobot, AFQ-Insight) along with a brief description of their respective roles and how they interoperate.*

As suggested, we have added a table (Table 1) with the different elements of the ecosystem. We find that Figure 1 allows better description of the interoperability between the different software components of the ecosystem, but this table certainly provides complementary and useful information for the readers.

*Conclusion: This is a well-executed and timely software paper that I believe will be of high value to the neuroimaging community. The above suggestions are offered only as potential ways to further increase its clarity and user-friendliness.*

Thanks again for these comments and assessment.

# 3 Reviewer #2:

*The authors present a suite of software tools for generating and analyzing tractometric profiles of major fiber bundles from diffusion MRI data. These tools can take preprocessed diffusion data, perform tractography, assign tracts to known anatomical bundles, compute summary profiles for each bundle based on diffusion properties or other characteristics, visualize these bundles and their quantitative profiles, and apply statistical and deep-learning models to these profiles to quantify group differences or predict subject phenotypes. Raw diffusion data can be handled through an integrated workflow within QSIprep, and the analytical tools can be applied to tractograms generated by other applications as well. The authors provide many example scripts to demonstrate how each step in the processing and analysis can be completed via python and R.*

Thank you for this summary of the article

*General comments: The set of tools presented are all impressive and certainly very useful for the neuroscientific community. The authors do present a compelling set of results to show that these tools are robust and neuroscientifically meaningful. However, much of this ecosystem has already been presented in the authors' previous publications, and much of this manuscript appears to be demonstrating aspects of the suite that have been previously demonstrated. It is not clear what portions of this manuscript describe new developments or additions to that work. Were the original tools not interoperable, and now are? Some new additions appear to be the neural network models and certain parallelization and acceleration options. The authors should be more explicit about what specifically is new here, and what is consolidated from existing work.*

While the present article builds upon previous work, it does present significant new developments, including version 2.0 of pyAFQ, which is a complete rewrite of the software that we published in 2021, as well as completely new approaches to statistical analysis and machine learning for tractometry. Interoperability among the tools is an important feature of the ecosystem, but is not the only point of novelty here. We added explanations of the significance of the present article in the Introduction (lines 41-46) and Discussion (lines 685-688), as well as clarifications in the Materials and Methods (lines 59-62, lines 282-285) of the main thrust of new developments in pyAFQ itself.

*Much of the emphasis appears to be on the improving the usability and interoperability of these tools. The authors should better guide readers and users through the examples on their "tractometry-ecosystem" github repository. Linking to this repository from the main tractometry.org website would be helpful. Upon arriving at the github README, it would be very helpful to be presented with a description of the available demos/notebooks, with direct links.*

We have revised the tractometry.org front-page to provide more useful information, including an overall explanation of the software ecosystem and its scientific context, as well as a fully rendered version of the examples that are available in this repository.

*Furthermore, presenting pre-baked outputs for these notebooks would help the user to better understand the "experience" of using the suite without the very lengthy startup/installation process.*

We have created a static version of the notebooks hosted on tractometry.org. The Code Ocean code capsule also has pre-baked outputs included in it. We have now clarified the documentation of the Code Ocean capsule, so that readers of the article and users of the code capsule have a better sense of how to use it, as it was indeed a bit opaque before.

*I was unable to successfully run the notebooks via either the "binder" or "codeocean" interfaces. The "binder" notebooks all encountered errors due to invalid file paths, and the "codeocean" site had no clear means or instructions to execute the actual notebooks.*

Thank you for pointing this out. The neurolibre/binder version had some technical issues running as we had hoped for, but we fixed these issues now and things should run as expected. We have now created a full executable version, which uses "pre-baked" files of already-run pipelines, so run-times should be rather short. For Code Ocean, we have also improved the documentation to explain how to run the notebooks through the Code Ocean interface.

> *Clarification: In line 275, can the authors clarify what they mean by 'We encoded the tract node as the "length dimension" in these one-dimensional networks'? In addition to this wording, I also think the related Fig S1 would be easier to understand if (e) showed several metrics or tracts, colored to match the column blocks in f-h, or otherwise maintained some more of the details from the similar Fig 1 in their 2021 paper.*

We have edited that section of the manuscript, using analogies to time-series and images, which we hope can help readers connect this process with the more common use-cases for these algorithms (lines 291-296). We have also completely revised the supplemental figure S1, using the same color scheme in the anatomy, and in the matrix tableau and network input. Thanks for this suggestion.

> *Minor: Missing reference in line 65.*

Thanks for spotting that. We added the cross-reference to the relevant section.

# 4 Additional reviewer comments

> *To further support the importance of quantitative analyses of tractography in the Introduction, please consider adding a citation to a recent review article:*
>
> *Zhang F, Daducci A, He Y, Schiavi S, Seguin C, Smith RE, Yeh CH, Zhao T, O'Donnell LJ. Quantitative mapping of the brain's structural connectivity using diffusion MRI tractography: A review. Neuroimage. 2022 Apr 1;249:118870.*

Thanks for suggesting this article. We have added a citation to it in the Introduction (line 7)
